# Diabetic Endothelium Dysfunction, Cardiovascular Complications, and Therapeutics

**DOI:** 10.1155/2016/5349801

**Published:** 2016-02-18

**Authors:** Yunzhou Dong, Yong Wu, Hyoung Chul Choi, Shuangxi Wang

**Affiliations:** ^1^Vascular Biology Program, Boston Children's Hospital, Harvard Medical School, Boston, MA 02115, USA; ^2^Department of Internal Medicine, Charles R. Drew University of Medicine and Science and the University of California, Los Angeles, CA 90059, USA; ^3^Department of Pharmacology, Yeungnam University, Daegu 712-749, Republic of Korea; ^4^The Key Laboratory of Cardiovascular Remodeling and Function Research, Qilu Hospital, Shandong University, Jinan 250012, China

Diabetes affects the life quality of a number of people largely through the cardiovascular complications [1]. Vascular endothelial cells play a major role in maintaining vascular homeostasis [2]. Dysfunction of the vascular endothelium is a critical factor in the pathogenesis of diabetic micro- and macrovascular diseases [2, 3]. The fundamental mechanism contributing to vascular disease, nephropathy, retinopathy, and neuropathy has yet to translate into effective therapeutics [4]. Uncovering novel mechanism governing endothelium dysfunction, new concepts about biological pathways involved in diabetic tissue injury, and identification of new therapeutics are of significance [5]. Circulating endothelial progenitor cells (EPCs) in diabetes are reduced and dysfunctional [6–8], suggesting EPC as a biomarker for diabetes and a prospective target for regenerative medicine [9].

Although significant strides have been made, the molecular mechanism of diabetic complications in vascular diseases and the effective treatment remain largely unknown. In this special issue, investigators have identified novel mechanism and therapeutics from their original research or review articles on the role of endothelial dysfunction in the etiology and pathogenesis of the micro- and macrovascular complications of diabetes, as well as therapeutic practice. Among the top gear, diabetes badly affects the function of retina and kidney. X. Cai and J. F. McGinnis systemically reviewed the mechanism of neovascularization, endoplasmic reticulum stress, inflammation, and aberrant angiogenesis in the pathogenesis of diabetic retinopathy and proposed some novel ideas for the DR treatment such as nanoceria, stem cells, miRNAs, and CRISPR/Cas9 technology. P. Li et al. demonstrated that inhibition of Na^+^/H^+^ exchanger 1 attenuates renal dysfunction by AGEs in rats, likely by the reduction of oxidative stress. In (pre)clinic studies, several groups have obtained promising outcomes in animal models or patients. W. Yu et al. suggested that Curcumin can improve cardiomyocyte function by inhibiting oxidative stress and apoptosis via the activation of Akt pathway; Z. Liu et al. proved that vitamin B6 prevents endothelial dysfunction, insulin resistance, and hepatic lipid accumulation in* ApoE*-null mouse model fed with high fat diet; R.-M. Cazeau et al. revealed that vitamin C and E administration can improve endothelium function in type 1 diabetic adolescents, while P. Yun et al. wrote an article showing that long-term administration of acarbose can effectively reduce the risk of the incidence of major adverse cardiovascular events in acute coronary syndrome patients, and possibly by the improvement of endothelium function. Further, S. Ghosal and B. Siniha reevaluated the use of Gliptins in cardiovascular patients in clinics, and M. Jamiolkowska et al. suggested that real-time continuous glucose monitoring (RT-CGM) may help in the detection of glycaemic variability, a newly recognized cardiovascular risk factor in adolescent type 1 patients.

As reported in recent years, endothelial progenitor cells (EPCs) are reduced in circulation [8, 9]; therefore, EPC homeostasis is critical in endothelial regeneration after injury. H.-Y. Tsai et al. demonstrated that Coenzyme Q10 can improve EPC function through AMP-kinase activation in diabetic condition, suggesting several benefits of Coenzyme Q10 for diabetic patients with cardiovascular complications such as atherosclerosis and hypertension.

Taken together, the articles in this special issue could further help researchers to understand the complexity of the diabetic complications in cardiovascular system and provide some new ideas to fight against diabetic cardiovascular complications.

*Yunzhou Dong* *Yunzhou Dong* *Yong Wu* *Yong Wu* *Hyoung Chul Choi* *Hyoung Chul Choi* *Shuangxi Wang* *Shuangxi Wang*
